# Timetable for oral prevention in childhood—developing dentition and oral habits: a current opinion

**DOI:** 10.1186/s40510-015-0107-8

**Published:** 2015-11-02

**Authors:** Alessandra Majorana, Elena Bardellini, Francesca Amadori, Giulio Conti, Antonella Polimeni

**Affiliations:** Dental Clinic, School of Dentistry, University of Brescia, P.le Spedali Civili 1, 25123 Brescia, Italy; Dental Clinic, IRCCS “Ca Granda-Ospedale Maggiore”, Department of Orthodontics, University of Milan, Milan, Italy; Department of Oral and Maxillo-facial Sciences, Pediatric Dentistry Unit, “Sapienza” University of Rome, Rome, Italy

## Abstract

As most of the etiologic factors of malocclusion are of genetic origin and thus cannot be prevented, environmental causative factors have become the focus for correction. Early interception of oral habits may be an important step in order to prevent occlusal disturbances in children. The identification of an abnormal habit and the assessment of its potential immediate and long-term effects on the dentition and potentially on the craniofacial complex should be made at an early stage. This paper focuses on the most common oral habits influencing dentofacial growth in childhood and management of these habits in the developing dentition.

## Background

The correct development of a stable, functional, and aesthetically acceptable occlusion is an integral component of comprehensive oral health care for all paediatric dental patients. Early diagnosis and successful treatment of developing malocclusion is pivotal in the development of occlusal harmony and function and dentofacial aesthetics [1, 2]. Dentists have the responsibility to recognize, diagnose, and manage or refer abnormalities in the developing dentition [3–5]. As most of the etiologic factors of malocclusion are of genetic origin and thus cannot be prevented, environmental aetiological factors may be the focus of attention. In this context, the early interception of oral habits may be integral in preventing occlusal disturbances [6–9].

### Clinical oral examination

The first oral examination is recommended at the time of the eruption of the first tooth and no later than 12 months of age [10–15]. The initial oral examination is best undertaken using the “knee-to-knee” approach, with the infant lying supine, supported by the parent on her/his lap, and the infant’s head lying on the dentist’s lap. A good light source, such as the dental operatory light, is essential for examination of the oral soft tissues, alveolar ridges, palate, and any erupted or erupting teeth. This visit represents an opportunity to discuss with the parents and caregivers the prevention of occlusal disturbances and about the importance to intercept oral habits. The developing dentition and occlusion should be monitored throughout eruption at regular clinical examinations [10–15].

### Oral habits

The habits of non-nutritive sucking, finger-sucking, tongue thrust swallow, and abnormal tongue position are the most common factors influencing dental development and potentially facial growth in childhood. The relationship between oral habits and unfavourable dental and facial development is associational rather than purely cause and effect [11, 14, 15]. Habits of sufficient frequency, duration, and intensity may be associated with dentoalveolar or skeletal deformations such as increased overjet, reduced overbite, posterior crossbite, or increased facial height. The duration of force is more important than its magnitude; the resting pressure from the lips, cheeks, and tongue has the greatest impact on tooth position, as these forces are maintained most of the time [11, 14, 16]. Non-nutritive sucking behaviours are considered normal in infants and young children. Prolonged non-nutritive sucking habits, however, have been associated with decreased maxillary arch width, increased overjet, decreased overbite, anterior open bite, and posterior crossbite [17]. According to Warren [18], there are significant differences in dental arch and occlusal relationships in pacifier users at 24 and 36 months compared with those that had stopped sucking by 12 months [15, 18]. Moreover, by age 2 to 5 years, a significant increase in overjet (>4 mm), open bite, and posterior crossbite in pacifier users was observed [17, 19].

### Principles of management

The frequency, duration, and intensity of the habit should be evaluated with identification of potentially harmful habits made as early as possible. Intervention to lead to habit cessation should be initiated if indicated [11, 17]. Both the Canadian Dental Association (CDA) and the American Dental Association (ADA) have similar guidelines on the appropriate use of pacifiers [17, 20]; more protracted use leads to a stronger association. The CDA recommends pacifiers over thumb sucking because it is easier for a parent to control the sucking habit. They advise against putting sugar, honey, or corn syrup on a soother because of the risk of caries induction and caution that a sucking habit should stop before the permanent teeth erupt. The ADA also advises parents who choose to use a pacifier to use a clean, unsweetened one. They state that although prolonged use can harm the teeth, it is easier to wean a child’s sucking habit from a pacifier than from a thumb [17, 21]. Early dental visits allow anticipatory guidance to help children to stop sucking habits by age 36 months or younger.

According to Italian Ministerial Guidelines [22, 23], it is recommended to encourage breastfeeding, in order to promote more normal jaw development. In fact, proper labial and lingual posture, adequate nasal breathing, and correct transverse diameter of the palate are related to natural breastfeeding. Artificial feeding may promote malocclusion, when combined with non-nutritive sucking or with rhinitis; in fact, bottle-fed children can develop sucking habits more frequently than others. Non-nutritive sucking, together with allergic rhinitis, seems to be the most important factor for the development of a posterior crossbite in children under 5 years [22, 23]. It is also recommended to discourage non-nutritive sucking at 2 years of age, in order to definitively stop the habit by 3 years of age. Beyond 3 years, non-nutritive sucking is implicated in malocclusions, such as anterior open bite, posterior crossbite, and Class II molar relationship. It is also recommended to monitor patients with low posture of the tongue and mouth breathing, in order to prevent dental open bite and excessive mandibular growth. Tongue thrusting and an abnormal tongue position may be associated with anterior open bite, speech difficulties, and anterior protrusion of the upper incisors [22, 23].

Research on the relationship between malocclusion and mouth breathing suggests that impaired nasal respiration may contribute to the development of increased facial height, anterior open bite, increased overjet, and narrow palate, although it is not the sole or even the major cause of these conditions [11, 24]. Mouth breathing children often present with skeletal II discrepancies, with transverse maxillary constriction, increased anterior facial height, and obtuse mandibular-maxillary planes angle. These findings may relate to the inferior tongue posture and hypotonia of facial muscles associated with mouth breathing [11, 24–26].

Management of an oral habit is indicated whenever the habit is associated with unfavourable dentofacial development or adverse effects on overall wellbeing or where there is a reasonable indication that the oral habit will result in unfavourable sequelae in the developing permanent dentition. Any treatment must be appropriate for the child’s development, comprehension, and ability to co-operate.

Habit treatment modalities include patient/parent counselling, behaviour modification techniques, myofunctional therapy, appliance therapy, or referral to other providers including, but not limited to, orthodontists, psychologists, myofunctional therapists, or otolaryngologists. Use of an appliance to manage oral habits is indicated only when the child wants to stop the habit and would benefit from a reminder [11]. Patients and their parents or caregivers should be informed regarding consequences of an oral habit. Parents may play a negative role in the correction of an oral habit as nagging or punishment may result in an increase in habit behaviours; change in the home environment may be necessary before a habit can be overcome.
